# What is this thing they call research?

**DOI:** 10.1093/jhps/hnx007

**Published:** 2017-03-27

**Authors:** Richard Villar

**Affiliations:** Editor-in-Chief, Journal of Hip Preservation Surgery

It was dinner, somewhere in the Middle East, me with my humanitarian hat on—there is a lot of that out there right now—and her my project boss. We were talking research, at least her desire the hospital should undertake some. I was being asked to advise.

My boss knew that research was a good thing, someone had most likely told her, but she had no idea what it involved. In her mind was the idea that research happened automatically, that anyone could do it and by some miracle a couple of weeks after a Nobel-winning brainwave, a scientific article might appear. I clearly had a long evening ahead.

Yet the fact is that research is a good thing. It is perhaps the best method of self-reflection there is. In my country, the United Kingdom, medicine is all about self-reflection at the moment. A doctor is obliged to reflect on this, ruminate on that, ponder on the other, indeed spend so much time perusing that there is a gradually decreasing time left over for our patients. There is even something they call a 360-degree feedback, where both colleagues and patients are asked what they think. Such is the dilemma with Government control of healthcare and the unwitting, maybe even intentional, side lining of doctors and their views.

Irrespective of the type of research—qualitative, quantitative, correlation/regression analysis, experimental, meta-analysis—at the editorial end I am frequently puzzled why so many hypotheses end up proving their point. The researcher has an idea, constructs a hypothesis and—Presto!—the hypothesis turns out correct. The problem of what to do with the negative result, the realization that so many of our journals appear to favour positive findings, is a phenomenon that was noticed many years ago and discourages authors from submitting negative results. Yet Pfeffer and Olsen in 2002 [1] had it right; however many confirming instances there are for a theory, it takes only one counter observation to falsify it. Heard of Gregor Mendel? His work on crosses of pea plants in the mid-19th Century led him to propose the First and Second Laws of Heredity, where certain traits of pea plants were determined by what he called factors but we now know as genes. No one believed him at the time. It took biomedicine another 40 years to repeat his work and the science of genetics was born. So, as an author, if you have that article which flies in the face of orthopaedic dogma, if your colleagues and other journals simply do not recognize its value, you may be years or decades before your time. Who knows? Send it to us and allow us to take a view. You never know. Your work may be precisely the topic we are seeking, so do send it along.

There is a fear, of course, that negative results are an admission of failure. Look at the regrettably established issue of failure to publish trial results. This is a very prevalent ethical breach with all its implications for patient care. Whether it is intentional or simply a reflection of a trial failing to adequately complete is clearly difficult to say, or to prove. The figures are astonishing. Chen *et al*. [2] looked at 4347 interventional clinical trials across 51 academic medical centres and found that the proportion of clinical trials published within 24 months of study completion ranged from 16.2 to 55.3%. Hardly impressive.

At *JHPS* we like to feel we are different. If your topic is hip preservation, in whatever form, then please, positive or negative, do submit your work to us. Each of us has researched extensively and is all too familiar with the tribulations and struggles of clinical research. Your reviewers are on side from the start. No promises but it is, at least, a happy beginning.

Turning to the last issue, number 3.4, I thought it was tremendous. As ever, I find it impossible to choose between so many excellent articles, but was especially attracted by the review of imaging for FAI by Albers *et al*., [3] very much a “go to” article for us all. And how about the feasibility study by Griffin *et al*. [4] for conducting a randomized controlled trial between arthroscopic hip surgery and conservative care? Now that, for sure, will create enormous debate.

And as for this issue, the first of 2017, might I suggest having a look at Grant *et al.* [5] who have addressed the issue of prehabilitation? Does it work or does it not? I will not spoil the surprise but, put it this way, I will most likely increase the frequency of prehabilitation having now read their article. Meanwhile if you need any encouragement to undertake hip arthroscopy with utmost care and caution do, please, read the article by Frandsen *et al.* [6] on traction-related problems after hip arthroscopy. They report that 74% of their patients had traction-related issues after hip arthroscopy. That is a lot of patients and an education to us all. Thank you to the authors for being so honest and helpful.

However, as always, I am spoilt for choice with this issue of *JHPS*. Enjoy every article and do let us know if you have any queries or concerns. And do also remember that article with negative results. We would be delighted to see it. A positive finding does not always mean success. 

My very best wishes to you all.
